# Height, weight, weight change and risk of breast cancer in Rio de Janeiro, Brazil

**DOI:** 10.1590/S1516-31802001000200005

**Published:** 2001-03-02

**Authors:** Anelise Bezerra de Vasconcelos, Gulnar Azevedo e Silva Mendonça, Rosely Sichieri

**Keywords:** Breast cancer, Body weight, Height, Body weight changes, Câncer de mama, Peso corporal, Estatura, Alterações ponderais

## Abstract

**CONTEXT:**

The relationship between body size and breast cancer still remains controversial in considering menopausal status.

**OBJECTIVE:**

To evaluate the association of height, weight and weight changes with breast cancer in the city of Rio de Janeiro, Brazil.

**DESIGN:**

Case-control study.

**SETTING:**

National Cancer Institute (INCA), Rio de Janeiro, Brazil, and State University of Rio de Janeiro (UERJ).

**SAMPLE:**

177 incidentcases of invasive breast cancer admitted to the main hospital of INCA between May 1995 and February 1996, and 377 controls recruited from among female visitors to the same hospital.

**MAIN MEASUREMENTS:**

Height and weight were measured and information on maximum weight, weight at ages 18 and 30 years, and potential risk factors were ascertained by interview at the hospital.

**RESULTS:**

Height was not related to risk of breast cancer among both pre and postmenopausal women. Nevertheless, women in this study were shorter than in studies that have found a positive association. Premenopausal women in the upper quartile of recent body mass index (BMI) and maximum BMI showed a reduced risk of breast cancer (P for trend ≤ 0.03). Weight loss between ages 18 and 30 years and from 18 years to present was also associated with breast cancer among premenopausal women.

**CONCLUSIONS:**

These findings may merely indicate the known association between leanness and breast cancer. Further studies should explore the role of weight loss on breast cancer risk.

## RESUMO

**CONTEXTO:**

A relação entre o peso corporal e o câncer de mama ainda permanece controversa ao ser considerado o estado menopausal.

**OBJETIVO:**

Avaliar a associação entre altura, peso e modificações no peso corporal com câncer de mama no município do Rio de Janeiro, Brasil.

**LOCAL:**

Instituto Nacional de Câncer (INCA), Rio de Janeiro, Brasil/ Universidade do Estado do Rio de Janeiro (UERJ).

**POPULAÇÃO DE ESTUDO:**

177 casos incidentes de câncer de mama invasivo admitidas no principal hospital do INCA, entre maio de 1995 e fevereiro de 1996, e 377 controles recrutados entre mulheres que visitavam pacientes no mesmo hospital.

**COLETA DE DADOS:**

Foram aferidos peso e altura e as informações sobre peso máximo, peso aos 18 e 30 anos, fatores de risco potenciais relacionados à história familiar de câncer de mama, história reprodutiva e escolaridade foram obtidas através de entrevista realizada no próprio hospital.

**RESULTADOS:**

A altura não se mostrou relacionada ao risco de câncer de mama entre as mulheres pré e pós-menopausa; entretanto, as mulheres estudadas apresentaram estaturas inferiores às relatadas em estudos que reportaram associação. Entre as mulheres na pré-menopausa o índice de massa corporal (IMC) recente e o IMC máximo associou-se negativamente ao risco de câncer de mama (P da tendência ≤ 0,03). Perda de peso entre os 18 e 30 anos de idade e entre os 18 anos e a idade presente foi associada com o aparecimento de câncer de mama antes da menopausa.

**CONCLUSÕES:**

Esses achados podem indicar, meramente, a conhecida associação entre magreza e câncer de mama. Estudos futuros deveriam explorar o papel da perda de peso para o risco de câncer de mama.

## INTRODUCTION

Breast cancer was the primary cause of cancer mortality among Brazilian women in 1996, and accounted for almost 23% of all cancer deaths among women in the city of Rio de Janeiro.^1^ Environmental factors, related to lifestyle and diet, have been implicated in the etiology of breast cancer.^2^

Adult stature is determined by parents’ stature, and is also an indicator of environmental factors such as energy intake during childhood and adolescence, especially in developing countries.^3^ As breast malignant neoplasm has a long latency period, the influence of nutritional factors during childhood and adolescence, may play an important role. Puberty is characterized by intense cell mitosis and tissue growth, with increases in height and weight, and the development of breast tissue. The influence of the sex steroids as mediators of growth, as well as risk factors for breast cancer is well known.^4-6^

Most case-control studies^7-11^ and cohort studies,^12-15^ but not all of them,^16-20^ have shown a positive association between height and breast cancer. Stature and breast size are positively related to the number of mammary cells, which are greatly influenced by energy intake and estrogen activity.^21^ Girls that experienced early menarche are likely to be taller and overweight.^22^

The influence of recent body weight on the risk of breast cancer appears to be controversial in relation to menopausal status. There is increasing evidence that obesity at the time of diagnosis and weight gain since youth increase the risk of breast cancer among postmenopausal women,^7,8,11,16,19,23-25^ although not confirmed by some other studies.^15,18,20^ However, obesity has been shown to be a protective factor for breast cancer among premenopausal women in many studies,^5,7,9,11,14,19,26-28^ but not all.^20^

Over the last 20 years, the height distribution and the prevalence of overweight in the Brazilian population has been increasing,^29-31^ which indicates that studies carried out in this population may be appropriate for investigating the association between body size and breast cancer.

This study evaluates the association of height, weight and weight changes with breast cancer, in a case-control study carried out in the city of Rio de Janeiro, Brazil, originally designed for testing the association between pesticides and other risk factors and breast cancer.^32^

## METHODS

Women admitted to the main hospital of the National Cancer Institute (INCA, located in Rio de Janeiro), between May 1995 and February 1996 with a presumptive diagnosis of breast cancer made in the preceding 6 months were selected. Control subjects were identified from among female visitors to the same hospital during the same period of time. Cases and controls had been resident in the metropolitan region of Rio de Janeiro for at least 6 months and were aged up to 75 years. In the control group, women with a personal history of breast cancer were excluded. Controls were frequency-matched to cases according to 5-year age groups. From all the eligible subjects, 95 (21.3%) of the controls and 2 cases refused to participate. Thus, 177 women with invasive breast carcinoma and 377 controls were included in the study. Among the controls, only 27 women were visiting relatives (first and second degree) with breast cancer.

Cases and controls were interviewed at the hospital to obtain detailed information on demographic data, family history of breast cancer, menstrual and reproductive history and dietary habits and previous weight, including weight at ages 18 and 30 years and the maximum weight during their lifetime, excluding pregnancy periods. Cases were interviewed prior to surgical procedures to minimize the possible influence of knowledge of disease status. Anthropometric measurements were taken after the interview.

Height, current body mass index (BMI) (kg/m^2^), BMI at ages 18 and 30 years, and maximum BMI, were categorized into specific quartiles for premenopausal and postmenopausal women and also treated as continuous variables. Weight changes since an early age were assessed by tertiles of weight gain, whereas weight loss was considered a fourth category. The single case aged 30 years was excluded from the analysis of weight loss from 18 to 30 years. Non-response was greater for weight between ages 18 and 30 years, but it was less than 17%. For maximum weight, the non-response was only 3.2%.

The odds ratios (OR) and 95 percent confidence intervals (95% CI) were calculated based on unconditional logistics regression adjusted for potential confounders, including age, age at menarche, parity, family history for breast cancer and education. Models were further adjusted for age at first full-term pregnancy and also smoking habits.

A reliability analysis of reported weights and height compared the current reported values with the measured ones.

## RESULTS

Cases and controls were similar according to education level (P = 0.74). Cases were more likely to be nulliparous, or had low parity, or experienced their first birth after 30 years and had early menarche (Table 1).

Height was not associated with breast cancer risk among pre and postmenopausal women. However, a non-significant trend of increasing risk with increasing height was observed among postmenopausal women (Table 2). A trend towards increasing height in more recent cohorts of women is shown, as premenopausal women were 4 cm taller than postmenopausal women (P < 0.05), and they also experienced earlier menarche (median of 12.82 years vs. 13.09 years).

Among premenopausal women, breast cancer was negatively associated with current BMI (χ^2^ for linear trend: P = 0.03) (Table 2) and also with maximum BMI during life. Premenopausal women in the fourth quartile of the maximum BMI during life had a very low risk of breast cancer (OR = 0.15; χ^2^ for linear trend: P = 0.008) compared to women in the lowest category (Table 3). For postmenopausal women, no association was observed for any of the body size measurements.

Premenopausal women who experienced weight loss between ages 18 and 30 years and from 18 years until the present were at high risk of breast cancer compared to those in the highest tertile of weight gain (Table 4). Even after adjusting for smoking (data not shown) the significant association with weight change remained. Weight loss between ages 18 and 30 years ranged from 1 to 18 kg among premenopausal women. Among those women that lost weight between ages 18-30, no case was diagnosed before 33 years of age. Considering as weight loss only those women who lost more than 3 kg, the association was kept but it was not significant (P = 0.12) (data not shown). Premenopausal women who had lost weight between ages 18 and 30 years were on average 6 kg leaner at 30 years compared to their weight at 18 years.

**Table 1:** Distribution of cases and controls according to age, educational level, parity, age at first full-term pregnancy and family history of breast cancer

Risk factors	Cases		Controls		P^a^
	n	Mean/%	n	Mean/%	
Age, years	177	56.9	377	56.5	0.71
Education level					
Illiterate	22	12.4	46	12.2	
Elementary school	74	41.8	179	47.5	
High school	67	37.8	127	33.6	
College	14	7.9	25	6.6	
					0.74
Age at menarche, years	177	12.9	374	13.8	0.31
Parity					
Nulliparous	32	18.1	47	12.5	
1 to 2	64	36.2	147	39.0	
3 to 4	51	28.8	106	28.1	
≥ 5	30	16.9	77	20.4	
					0.29
Age at first full-term pregnancy					
< 20 years	43	29.7	95	28.8	
20 to 29 years	79	54.5	199	60.3	
≥ 30 years	23	15.9	36	10.9	
					0.27
Family history of breast cancer,%^b^					
Not mentioned	153	90.0	339	90.2	
0.1 to 24.9	6	3.5	10	2.7	
25 to 49.9	5	2.9	18 4.	8	
≥ 50	6	3.5	9	2.4	
					0.61

a - As determined by t-test or χ2; b - Number of cases among first degree relatives/female family size (mother+number of sisters).

**Table 2:** Distribution of cases and controls according to height and current BMI and associated odds ratios for breast cancer among pre and postmenopausal women

Variables	Cases n	Controls n	Quartiles OR^a^	95% CI
**Premenopausal women**				
*Height^b^*				
<152cm	8	18	1.00	-
152-155 cm	9	16	1.54	0.44 to 5.32
156-159 cm	10	22	1.18	0.33 to 3.86
≥160 cm	14	20	1.54	0.46 to 5.15
*χ*^2^ for trend			P = *0.52*	
*Current BMI(kg/m^2^)*				
< 22.79	13	16	1.00	-
22.79-26.47	11	18	0.74	0.24 to 2.21
26.48-30.23	10	20	0.55	0.18 to 1.68
≥30.23	7	22	0.25	0.07 to 0.93
*χ*^2^ for trend			P = *0.03*	
**Postmenopausal women**				
*Height^b^*				
<149 cm	25	65	1.00	-
149-151 cm	20	53	0.93	0.45 to 1.92
152-156 cm	47	79	1.39	0.74 to 2.58
≥157 cm	36	65	1.29	0.67 to 2.50
*χ*^2^ for trend			P = *0.28*	
*Current BMI(kg/m^2^)*				
<24.55	38	60	1.00	-
24.55-27.64	29	69	0.61	0.33 to 1.14
27.65-30.79	35	64	0.84	0.46 to 1.53
≥30.80	29	69	0.61	0.33 to 1.14
*χ*^2^ for trend			P = *0.24*	

^a^Adjusted for age, parity, age at menarche, family history of breast cancer and education; ^b^Also adjusted for recent weight residuals.

The risk of breast cancer for all anthropometric variables did not change significantly after adjusting for age at first full-term pregnancy. This was not included in all models because about 15% were nulliparous.

There was a high correlation between reported and measured weight. The intra-class correlation for weight was 0.98 for cases, and 0.95 for controls, whereas for height, the intraclass correlation was 0.92 for cases and 0.94 for controls.

## DISCUSSION

In the present study, cases were more likely to be nulliparous, had low parity, experienced their first birth after 30 years of age, and had early menarche, in agreement with current literature, although no statistically significant differences were observed (Table 1). In addition, the family history of breast cancer was not different between cases and controls. Mendonça et al,^32^ analyzing data from the same case-control study, reported a positive significant association with a family history of breast cancer, after excluding the twenty-seven controls visiting first or second degree relatives with breast cancer, which suggests that the lack of association may be attributed to overmatching. As family history of breast cancer was one of the variables selected for the multivariate analysis, we decided not to exclude those related controls.

Height was not significantly associated with increased risk of breast cancer among pre and postmenopausal women. In studies carried out in developed countries, taller women seem to be at high risk of breast cancer,^7-13^ but other studies have not found this association.^14,15^ Results of studies on laboratory animals support the hypothesis that early energy restriction may decelerate height, delaying the onset of sexual maturation, and reducing the oncogene expression and tumor growth.^4,33,34^

The lack of association between height and breast cancer in this study may be due to the fact that the Brazilian population is still shorter than the population in developed countries even with the increasing height that has been shown in Brazilian National Surveys.^28,29^ The fourth quartile value for height, for both pre and postmenopausal women in our study, is very close to the reference category in three prospective studies carried out in developed countries.^14,15,19^ Also, premenopausal women in our study were four centimeters taller than postmenopausal women, and experienced earlier menarche, indicating an improvement in nutrition during growth periods over more recent years.

Our data agree with other findings^7,9,11,14,19,25,28^ that obesity is inversely related to breast cancer risk among premenopausal women. Obese young women are more likely to have anovulatory and longer menstrual cycles, leading to low estrogen exposures of the breast cells^5,27^ and a decreased mitosis rate for the mammary tissue.^27^ Obese young women also have low levels of progesterone, which seems to maximize estrogen action during the luteal phase.^35^ There is also evidence that the body mass index before menopause is inversely related to serum level estradiol.^36^ Leanness among young women is associated with high estrogen activity.^36-38^

**Table 3:** Distribution of cases and controls according to BMI at early ages and maximum BMI and associated odds ratios for breast cancer among pre and postmenopausal women

Variables	Cases (n)	Controls (n)	Quartiles OR^a^	95% CI
**Premenopausal women**				
*BMI at age 18 years, kg/m^2^*				
*<=* 18.37	12	14	1.00	-
18.38-20.54	8	18	0.52	0.16 to 1.69
20.55-22.80	7	19	0.43	0.12 to 1.48
>=22.81	9	18	0.50	0.15 to 1.64
*χ^2^* for trend		P = *0.35*		
*BMI at age 30 years, kg/m^2^*				
<=21.01	8	20	1.00	-
21.02-23.76	11	18	1.45	0.46 to 4.53
23.77-26.30	9	20	0.99	0.29 to 3.35
>=26.31	6	22	0.35	0.08 to 1.54
*χ*^2^ for trend		P = *0.17*		
*Maximum BMI, kg/m^2^*				
<=24.27	14	18	1.00	-
24.28-27.18	9	22	0.46	0.15 to 1.39
27.19-30.47	9	23	0.45	0.15 to 1.35
>=30.48	6	26	0.15	0.03 to 0.60
*χ*^2^ for trend		P = *0.008*		ï
**Postmenopausal women**				
*BMI at age 18 years, kg/m^2^*				
<=18.58	25	61	1.00	-
18.59-20.70	31	55	1.74	0.89 to 3.42
20.71-23.30	33	55	1.71	0.88 to 3.32
>=23.31	19	67	0.84	0.41 to 1.74
*χ*^2^ for trend		P = *0.69*		
*BMI at age 30 years, kg/m^2^*				
<=20.80	30	54	1.00	-
20.81-22.91	22	64	0.71	0.36 to 1.43
22.92-25.21	32	54	1.17	0.61 to 2.25
>=25.22	25	60	0.82	0.42 to 1.61
*χ*^2^ for trend		P = *0.96*		
*Maximum BMI, skg/m^2^*				
<=26.01	34	64	1.00	-
26.02-29.14	28	70	0.70	0.38 to 1.32
29.15-32.79	30	69	0.79	0.43 to 1.48
>=32.80	33	67	0.92	0.50 to 1.70
*χ*^2^ for trend		P = *0.92*		

*Adjusted for age, parity, age at menarche, family history of breast cancer and education.*

**Table 4:** Distribution of cases and controls according to weight change and associated odds ratios for breast cancer among pre and postmenopausal

	women							
		Premenopausal women				Postmenopausal women		
Variables	Cases (n)	Controls (n)	OR^a^	95% CI	Cases (n)	Controls (n)	OR^a^	95% CI
*Weight change since age 18 years (kg)^b^*								
> 22.3kg	7	21	1.00	-	31	72	1.00	-
13.11-22.3kg	9	21	1.73	0.43 to 6.93	38	67	1.39	0.75 to 2.59
0-13.10 kg	16	24	2.93	0.85 to 10.02	28	66	1.24	0.62 to 2.50
Weight loss	4	3	16.65	1.75 to 157.80	12	21	2.05	0.75 to 5.59
*χ*^2^ for trend			P = *0.01*				P = *0.24*	
*Weight from 18 to 30 years (kg)^b^*								
> 10kg	8	22	1.00	-	14	37	1.00	-
5.1-10.0kg	8	17	1.63	0.37 to 7.22	31	56	1.96	0.86 to 4.48
0-5.0 kg	12	36	1.20	0.34 to 4.23	50	108	1.68	0.75 to 3.77
Weight loss	5	3	29.02	2.39 to 351.19	8	20	1.57	0.49 to 5.03
*χ*^2^ for trend			P = *0.16*				P = *0.44*	
*Weight change since age 30 years (kg)^c^*								
> 16.2kg	7	14	1.00	-	37	58	1.00	-
9.1-16.2kg	19	5	0.48	0.10 to 2.29	29	62	0.79	0.42 to 1.50
0-9.0 kg	18	19	1.48	0.37 to 5.81	25	65	0.67	0.34 to 1.32
Weight loss	4	14	0.72	0.14 to 3.66	19	35	0.98	0.44 to 2.17
*χ*^2^ for trend			P = *0.71*				P = *0.67*	

In contrast to most studies,^7,8,11,16,18,19,24^ recent obesity and weight gain were not associated with breast cancer risk among postmenopausal women. Less variability of BMI among Brazilian women may explain this lack of association. The third and fourth quartiles of BMI in our study correspond to the fifth quintile in a population based case-control study carried out in the USA which showed evident association above the fifth decile of BMI.^7^ After menopause, obese women have a high level of serum estrogen as a consequence of the conversion of androsteredione to estrone in the adipose tissue, and also due to decreasing concentration of the Sex Hormone Binding Globulin (SHBG), that increases the serum free-estrogen.^24,27^ The high production of estrogen may promote tumor growth, although the development of tumor growth depends on whether breast cell damage was replicated during the intense breast development, i.e., childhood and puberty period. As postmenopausal women in our study were shorter, they may have had a reduction in the mammary gland mass.^21^

We did find a strong positive association for weight loss from age 18 to 30 and for weight loss from age 18 years until the present among premenopausal women. Weight loss was not a sign of preclinical disease, since the case diagnosed before 33 years (only one woman) was not included in the analysis. These weight losses may also not be due to smoking, as the results did not change after adjusting for smoking. To our knowledge, there are few studies relating weight loss to breast cancer risk. Recently, a casecontrol study reported a slightly non-significant reduced risk for weight loss from age 18 years until five years before the beginning of that study.^4^

A cross-sectional study of 76 college women that underwent a restricted diet had a reduction in menstrual cycle length compared to those nondieters,^39^ in accordance with other study.^40^ Women with a short menstrual cycle may be at high risk of breast cancer because they spend relatively more time in the luteal phase, characterized by breast cell mitosis, than women with longer menstrual cycles.^41,42^ Harlow et al.^43^ showed among 166 college women aged 17 to 19 years that overweight women were more likely to have a menstrual cycle longer than 43 days, whereas women who experienced loss of weight tended to reduce the length of cycles. Thus, weight loss may be associated with premenopausal breast cancer by shortening menstrual cycle length.

There was no evidence of a differential misclassification among cases and controls related to reported weights and height in this study. The correlation coefficients for measured and self-reported weight and stature were up to 0.90 for both groups. In addition, error in reported body sizes would bias our risk estimations toward the null value.^44^

## CONCLUSION

Our findings showed a strong positive association for weight loss from age 18 to 30 and for weight loss from age 18 years until the present among premenopausal women, suggesting that obesity was inversely related to breast cancer risk among these women. These findings, with highest risk among those women with weight loss, may merely indicate the known association between leanness and breast cancer. Nevertheless, the small number of women losing weight in our study, as well as in many studies of breast cancer, makes it hard to confirm this possibility. Further studies should explore the role of weight loss on breast cancer risk.

## References

[1] [DATASUS] Brasil. Ministério da Saúde (1998). Sistema Único de Saúde (SUS). Sistema de Informação Hospitalar.

[2] Ziegler RG, Hoover RN, Pike MC, Hildesheim A, Nomura AM, West DW (1993). Migration patterns and breast cancer risk in Asian-American women. J Natl Cancer Inst.

[3] Sichieri R, Taddei JA, Everhart JE Influence of parental height and sociodemographic factors on adolescent height in Brazil. J Adol Health.

[4] Micozzi MS, Moon TE (1992). Macronutrients: investigating their role in cancer.

[5] Ballard-Barbash R (1994). Anthropometry and breast cancer: body size: a moving target. Cancer.

[6] Saggese G, Bertelloni S, Baroncelli GI (1997). Sex steroids and the acquisition of bone mass. Horm Res.

[7] Trentham-Dietz A, Newcomb PA, Storer BE (1997). Body size and risk of breast cancer. Am J Epidemiol.

[8] Swanson CA, Brinton LA, Taylor PR (1989). Body size and breast cancer risk assessed in women participating in the Breast Cancer Detection Demonstration Project. Am J Epidemiol.

[9] Swanson CA, Coates RJ, Schoenberg JB (1996). Body size and breast cancer risk among women under age 45 years. Am J Epidemiol.

[10] Minnist S, Pietinin P, Palmgren J, Eskelinen M, Uusitupa M (1996). Body size indicators and risk of breast cancer according to menopause and estrogen-receptor status. Int J Cancer.

[11] Ziegler RG, Hoover RN, Nomura AMY (1996). Relative weight, weight change, height and breast cancer risk in Asian-American women. J Natl Cancer Inst.

[12] Albanes D, Jones DY, Shatzkin A, Micozzi MS, Taylor PR (1988). Adult stature and risk of breast cancer. Cancer Research.

[13] Hunter DJ, Willett WC (1993). Diet, body size and breast cancer. Epidemiol Rev.

[14] Vatten LJ, Kvinnsland S (1992). Prospective study of height, body mass index and risk of breast cancer. Acta Oncol.

[15] van den Brandt PA, Dirx MJ, Ronckers CM, van den Hoogen P, Goldbohm RA (1997). Height, weight, weight change and postmenopausal breast cancer: The Netherlands Cohort Study. Cancer Causes Control.

[16] Chie WC, Li C, Chang KJ (1998). Body size as a factor in different ages and breast cancer risk in Taiwan. Anticancer Res.

[17] Tavani A, Braga C, La Vecchia C (1998). Height and breast cancer risk. Eur J Cancer.

[18] Folsom AR, Kaye SA, Prineas RJ (1990). Increased incidence of carcinoma of the breast associated with abdominal adiposity in postmenopausal women. Am J Epidemiol.

[19] London SJ, Colditz GA, Stampfer JJ (1989). Prospective study of relative weight, height and risk of breast cancer. J Am Med Assoc.

[20] Kaaks R, van Noord PAH, Tonkelaar I (1998). Breast-cancer incidence in relation to height, weight and body-fat distribution in Dutch "DOM’ cohort. Int J Cancer.

[21] Trichopoulos D, Lipman RD (1992). Mammary gland mass and breast cancer risk. Epidemiology.

[22] Apter D (1996). Hormonal events during female puberty in relation to breast cancer risk. Eur J Cancer Prev.

[23] Magnusson C, Baron J, Persson I (1998). Body size in different periods of life and breast cancer risk in postmenopausal women. Int J Cancer.

[24] Cauley JA, Gutai JP, Kuller LH, LeDonne D, Powell JG (1989). The epidemiology of serum sex hormones in postmenopausal women. Am J Epidemiol.

[25] Huang Z, Hankinson SE, Colditz GA (1997). Dual effects of weight and weight gain on breast cancer risk. JAMA.

[26] Ursin G, Longnecker MP, Haile RW, Greenland SA (1995). A meta-analysis of body mass index and risk of premenopausal breast cancer. Epidemiology.

[27] Hulka BS, Liu ET, Lininger RA (1994). Steroid hormones and risk of breast cancer. Cancer.

[28] Peacock SL., White E, Daling JR, Voigt LF, Malone KE (1999). Relation between obesity and breast cancer in young women. Am J Epidemiol.

[29] Fundação Instituto Brasileiro de Geografia e Estatística (1977). Estudo Nacional da Despesa Familiar – ENDEF.

[30] Coitinho DC, Leão MM, Recine E, Sichieri R (1991). Condições nutricionais da população brasileira. Pesquisa Nacional de Saúde e Nutrição.

[31] Sichieri R (1998). Epidemiologia obesidade.

[32] Mendonça GAS, Eluf-Neto J, Andrada-Scarpa MJ (1999). Organochlorines and breast cancer: a case-control study in Brazil. Int J. Cancer.

[33] Rogers AE (1997). Diet and breast cancer: studies in laboratory animals. J Nutrition.

[34] Klurfeld DM, Welsch CB, Lloyd LM (1989). Inhibition of DMBA – induced mammary tumorogenesis by caloric restriction in rats fed high fat diets. Int J Cancer.

[35] Key TJA, Pike MC (1988). The role of oestrogens and progestagens in the epidemiology and prevention of breast cancer. Eur J Cancer Clin Oncol.

[36] Potischman N, Swanson CA, Sitteri (1996). Reversal of relation between body mass and endogenous estrogen concentrations with menopausal status. J Natl Cancer Inst.

[37] Gerber M, Letters to the Editor (1997). Body size and breast cancer risk among women under age 45 years. Am J Epidemiol.

[38] Semmens J, Rouse I, Beilin LJ (1984). Masarei JRL Relationship of plasma HDL-cholesterol to testosterone, estradiol, and Sex-Hormone-Binding Globulin levels in men and women. Metabolism.

[39] Rock CL, Gorenflo DW, Drewnowski A, Demitrack MA (1996). Nutritional characteristics, eating pathology, and hormonal status in young women. Am J Clin Nutr.

[40] Barr SI, Janelle C, Prior JC (1994). Vegetarian vs. non-vegetarian diets, dietary restraint, and subclinical ovulatory disturbances: prospective 6-month study. Am J Clin Nutr.

[41] Whelan EA, Sandler DP, Root JL, Smith KR, Weinberg CR (1994). Menstrual cycle patterns and risk of breast cancer. Am J Epidemiol.

[42] Yuan J, Yu MC, Ross RK, Gao Y, Henderson BE (1988). Risk factors for breast cancer in Chinese women in Shanghai. Cancer Res.

[43] Harlow SD, Matanoski GM (1991). The association between weight, physical activity, and stress and variation in the length of the menstrual cycle. Am J Epidemiol.

[44] Raphael K (1987). Recall Bias: A proposal for assessment and controls. International Journal of Epidemiology.

